# Epigenetic Regulation of Organ Regeneration in Zebrafish

**DOI:** 10.3390/jcdd5040057

**Published:** 2018-12-14

**Authors:** Xiaojun Zhu, Chenglu Xiao, Jing-Wei Xiong

**Affiliations:** Beijing Key Laboratory of Cardiometabolic Molecular Medicine, Institute of Molecular Medicine, and State Key Laboratory of Natural and Biomimetic Drugs, Peking University, Beijing 100871, China; chengluxiao2016@163.com

**Keywords:** regeneration, zebrafish, DNA methylation, histone modification, SWI/SNF complex, miRNA

## Abstract

The zebrafish is broadly used for investigating de novo organ regeneration, because of its strong regenerative potential. Over the past two decades of intense study, significant advances have been made in identifying both the regenerative cell sources and molecular signaling pathways in a variety of organs in adult zebrafish. Epigenetic regulation has gradually moved into the center-stage of this research area, aided by comprehensive work demonstrating that DNA methylation, histone modifications, chromatin remodeling complexes, and microRNAs are essential for organ regeneration. Here, we present a brief review of how these epigenetic components are induced upon injury, and how they are involved in sophisticated organ regeneration. In addition, we highlight several prospective research directions and their potential implications for regenerative medicine.

## 1. Introduction

**Figure 1:**
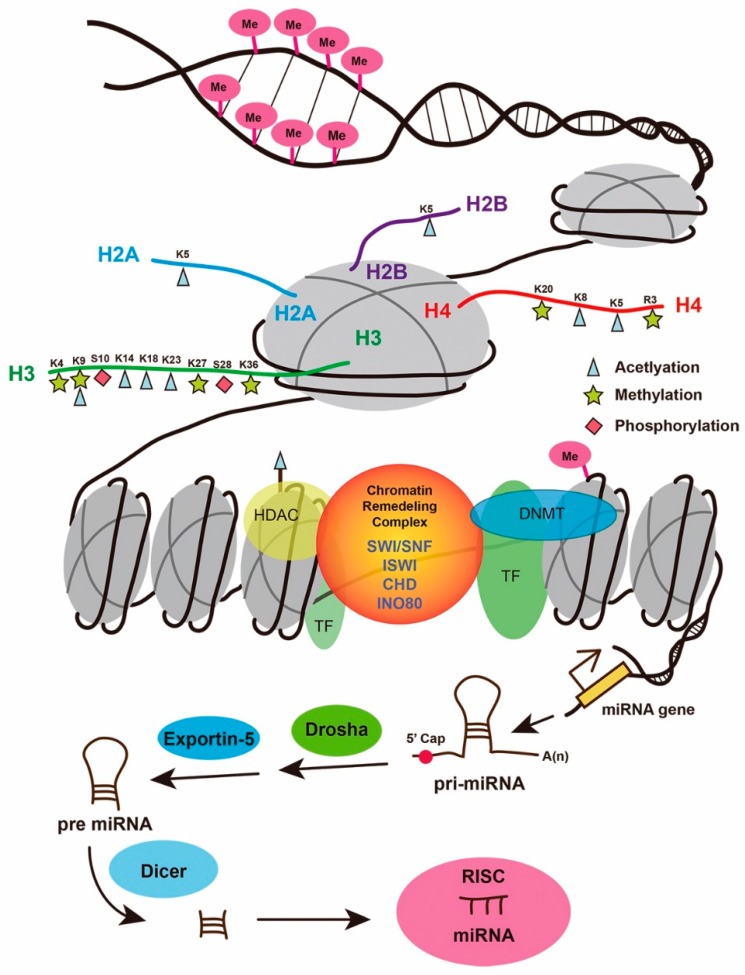
Schematic illustration of epigenetic regulation. Epigenetic regulation mainly comprises DNA methylation, histone modifications, chromatin remodeling complexes, and non-coding RNAs. CHD—CHD chromatin-remodeling complexes; K4, 9, 20, 27, 36, and R3—histone lysine (K) or arginine (R) methylation; K5, 8, 14, 18, and 23—histone lysine (K) acetylation; INO80—INO80 chromatin-remodeling complexes; ISWI—SWI-like ATP-dependent chromatin-remodeling complexes; miRNA—microRNA; Me—DNA methylation; pri-miRNA—primary miRNA; pre-miRNA—precursor miRNA; S10 and 28—histone phosphorylation; SWI/SNF—SWItch/sucrose nonfermentable complex; TF—transcription factor.

The regeneration of lost cells or tissues after injury will revolutionize modern medicine, especially for diseases with high morbidity and mortality, such as myocardial infarction, stroke, and degenerative neurological diseases. During organ regeneration, injury induces a series of biological responses, including inflammation, cell dedifferentiation, proliferation, and redifferentiation. These processes engage with both genetic and epigenetic changes that trigger transcriptional and translational activation or repression at a molecular level. Epigenetic regulation comprises multiple components, such as DNA methylation and hydroxymethylation; histone modifications, including methylation, acetylation, and phosphorylation; chromatin remodeling complexes; and non-coding RNAs (Figure 1) [1]. While epigenetic modifications in a variety of biological processes, including early embryonic and organ development, have been well reviewed, here, we present a brief summary of how epigenetic factors regulate organ regeneration in zebrafish. The zebrafish is well-recognized as a major vertebrate organism for studying organ regeneration, so an elucidation of the underlying mechanisms will likely provide novel strategies and technologies for enhancing organ repair and regeneration in humans.

Because of their strong regenerative potential, most zebrafish organs are the subject of ongoing work, including the tail fin, heart, pancreas, liver, retina, brain, and spinal cord [2]. Major progress is reflected in a number of novel insights into both the cellular and molecular mechanisms underlying organ regeneration. Here, we present a brief introduction to these mechanisms in several organs in zebrafish.

Retinal regeneration: After retinal injury, the Müller glia rarely re-enters the cell cycle and regenerates new neurons, resulting in reactive gliosis and scarring in mammals. In contrast, the adult zebrafish retina regenerates after different kinds of injury, including surgical, chemical, and light-induced lesions, or genetically-targeted, metranidozole-mediated cell ablation [2]. Lineage-tracing has identified the Müller glia as the source of regenerated neurons [3]. After injury, the Müller glia undergoes transient dedifferentiation, asymmetric division, and redifferentiation. Their dedifferentiation is indicated by the increased expression of retinal progenitors and immature Müller glial markers, as well as by decreased differentiation markers. Reprogrammed Müller glia divide and generate the retinal progenitor cells, which are capable of regenerating all major types of retinal neurons [3].

Cardiac regeneration: Adult zebrafish can survive and fully regenerate the heart after an injury, such as apex amputation or cryoinjury [4,5,6]. After apex amputation, blood clots form immediately to cover the wound. From the first few days post amputation (dpa), fibrins accumulate in the injured area, cardiomyocytes then proliferate and penetrate into the injured area, and new cardiomyocytes eventually replace the blood clots. The whole process takes 30–60 days. Genetic lineage-tracing has shown that cardiomyocytes regenerate from the dedifferentiation and proliferation of existing cardiomyocytes [7,8]. Mechanistic studies have mainly focused on how epicardial, endocardial, and myocardial signaling pathways regulate the regeneration of cardiomyocytes.

Fin regeneration: The caudal fin regenerates completely within 2–4 weeks after amputation. Regeneration consists of at least five steps, namely: wound healing, wound epithelium establishment, blastema formation, outgrowth, and termination [9,10]. After amputation, a specialized wound epithelium and the blastema are formed [11]. The wound epidermis functions as a physical barrier to protect the internal fin tissues, and supplies a niche-like environment for the blastema [11]. The blastema contains undifferentiated stem cell-like cells and is highly proliferative and essential for fin regeneration [12]. Genetic lineage-tracing has revealed that both the zebrafish caudal fin and salamander limb cell-types regenerate from themselves or their own progenitors, but not by transdifferentiation [13,14,15,16]. New tissue forms by proliferation and progressively re-differentiates into a mature fin fold. The Notch, Wnt (wingless/integrated), Bone Morphogenetic Protein, and Sonic Hedgehog signaling pathways are essential for fin regeneration and growth [17].

Central nervous system regeneration: The zebrafish is able to fully regenerate lost neurons after either a traumatic brain lesion or the complete transection of the spinal cord [18,19]. After injury, the inflammatory response is limited to 1–7 days in a telencephalon injury, or 15–30 days in spinal transection. Radial glia cells serve as neural precursor cells and give rise to both neurons and glia [19]. Cysteinyl leukotriene signaling and Gata3 are essential for regeneration in the adult zebrafish brain [20,21]. In the spinal cord, radial glia give rise to a glial bridge that guides axonal regrowth, and no glial scar develops during regeneration. The connective tissue growth factor is both necessary and sufficient to initiate glial bridging and spinal cord regeneration in zebrafish [22].

There are several excellent reviews on the cellular and molecular mechanisms underlying the regeneration of the retina, heart, spinal cord, and fin [2,10,11,13,23,24,25,26,27]. In this article, we omit many classical works in this field, and pay particular attention to recent advances in the epigenetic regulation of tissue/organ regeneration in zebrafish.

## 2. Role of DNA Methylation in Organ Regeneration

Genomic DNA can be methylated at the fifth carbon position of cytosines in the 5′-cytosine-phosphate-guanine-3′ dinucleotide. Cytosine methylation is often inversely correlated with gene expression, although it sometimes has positive effects on transcriptional regulation [28]. In particular, DNA methylation plays a pivotal role in X-chromosome inactivation, gene silencing, genomic stability, and imprinting. Accumulating evidence has demonstrated their crucial functions during early embryogenesis, carcinogenesis, and aging [29,30]. The methylation and demethylation processes counter-balance each other to determine the status of the DNA methylation. DNA methyltransferases catalyze the addition of a methyl group to the 5-carbon of cytosine, generating 5-methylcytosine (5mC). Demethylation is a multistep process mediated by the ten-eleven translocation (TET) enzymes, which catalyze the conversion of 5mC to 5-hydroxymethylcytosine (5hmC), 5-formylcytosine (5fC), or 5-carboxylcytosine (5caC). The 5fC and 5caC can be excised by G/T-mismatch-specific thymine DNA glycosylase (TDG), resulting in apyrimidinic sites. These sites can be replaced by cytosine through base excision repair (BER). Besides these, activation-induced cytidine deaminase and APOBEC (apolipoprotein B mRNA editing enzyme, catalytic polypeptide-like) deaminate 5mC or 5hmC to form 5-methyluracil or 5-hydroxymethyluracil, which can be catalyzed to cytosine via the TDG/BER pathway [31,32].

While they are normally turned off in adult organs, a number of developmental signaling pathways are reactivated during their regeneration, leading to the hypothesis that the mechanisms known to regulate organ development are sometimes reused in the regeneration processes. The level of DNA methylation changes dynamically during embryogenesis. TET activity is required for retinal development in zebrafish. The *tet2^−/−^; tet3^−/−^* double mutant displays impaired terminal differentiation in retinal neurons [33]. The dnmt1 mutant has similar defects in hematopoiesis and in jaw, liver, exocrine pancreas, and eye development [34,35,36]. *Dnmt2*-knockdown results in defects in retina, liver, and brain development [37]. *Dnmt3* morphants display multiple abnormalities throughout embryogenesis, including defects in neurogenesis [38]. In addition, *Dnmt1* is unable to rescue the defects in the *dnmt3* morphants and vice versa [38]. These results suggest that DNA methylation is essential for organ development, in addition to its critical roles in early vertebrate embryogenesis.

During regeneration after either injury or resection of different organs, inflammation, cell dedifferentiation, and proliferation occur sequentially. The global status of DNA methylation changes during this process. The methylation status of the Xenopus elongation factor 1-α (ef1α):EGFP transgene fluctuates dynamically during fin and retina regeneration. This transgene is silenced in adult tissues, including the caudal fin and retina, but is re-activated after fin resection or retinal injury, consistent with the demethylation of the ef1α:EGFP transgene [39]. Ef1α:EGFP is expressed in the blastema after fin injury and in Müller glia, which are the major source of progenitor cells in the retina [39]. Together, these studies suggest that the DNA methylation status regulates gene silencing or activation during tissue regeneration [39]. Future studies are needed to address how DNA methylation is globally regulated and what types of regenerative genes are influenced by this modification during the regeneration of different organs.

Retinal regeneration: After retinal injury, the expression of genes associated with DNA methylation and demethylation is strongly or moderately induced, although the time at which each gene reaches its peak induction differs [40,41]. Most importantly, after the knockdown of *apobec2a* and *apobec2b*, which deaminate the cytosine and contribute to DNA demethylation, the proliferation and regeneration of the Müller glia in the retina and optic nerve are also blocked [41]. However, a later study has reported that the DNA methylation changes during regeneration are largely independent of the Apobec2 expression [40]. It remains unclear whether the Apobec2a and Apobec2b proteins regulate retinal regeneration via the signals independent of DNA methylation. The promoters of the pluripotency factors and some regeneration-associated genes remain hypomethylated in both Müller glia (MG) and their progenitor cells (MGPCs). On the other hand, the promoter and intragenic regions of the genes that are highly induced in MGPCs at 4 dpi are less methylated, suggesting that the induction of a gene expression is less likely attributable to, or inversely related to DNA demethylation. DNA demethylation may be required for, but not sufficient to drive MG reprogramming. During the transition from MGs to MGPCs, the genomic DNA methylation status undergoes dynamic changes; it predominates at 0–2 dpi, when MGPCs are forming. From 2 to 4 dpi, when MGPCs are expanding, the levels of de novo methylation increase [40]. Future studies are needed in order to address how the level of DNA methylation is functionally correlated with transcriptional regulation during organ regeneration in particular, and other biological processes in general.

Fin regeneration: After fin resection, DNA demethylation occurs in the dedifferentiated cells. The levels of 5mC and 5hmC in the blastema cells and cells adjacent to the amputation area decrease at 12 h post-amputation. The 5mC level in the blastema cells decreases at 30 h and is almost restored at 72 h, while the 5hmC level remains low, until it returns to the basal level at 14 days post-amputation, when regeneration is complete [42]. Some genes associated with DNA demethylation and repair, such as *gadd45* (growth arrest and DNA damage), are induced [42], and the *dnmt3aa* expression is evident in the blastema cells during fin regeneration after 72 h post-amputation [43]. These profiling studies strongly suggest that DNA methylation is dynamically down-regulated during regeneration, and returns to the basal level after regeneration is completed. The exact role of DNA methylation in fin regeneration warrants further investigation.

Briefly, DNA methylation is the key regulatory machinery during cell dedifferentiation and proliferation. Although global changes of DNA methylation during the regeneration of the fin and retina have been reported, this phenomenon has not been addressed in cardiac regeneration. Overall, the exact mechanisms that balance methylation and demethylation, the changes in the spatiotemporal profile of DNA methylation, and the genes targeted via the changes in DNA methylation during organ regeneration remain to be investigated.

## 3. Histone Modification and Gene Regulation in Organ Regeneration

Each nucleosome consists of an octamer with two copies of four different histone proteins (H2A, H2B, H3, and H4). It is well known that the histone tails can be chemically modified via acetylation, methylation, phosphorylation, or ubiquitylation, thus changing the conformation of chromatin and regulating the activation of transcription or inducing silence. The acetylation of lysine on histone tails is often associated with transcriptional activation. Histone methylation can either activate or inhibit gene transcription, depending on which amino-acid is methylated.

Compared with other epigenetic modifications, there are very limited studies on the role of histone modifications in injury-induced organ regeneration in zebrafish. During fin regeneration, H3K9me2, H4K20me3, H3K4me3, and H3K14ac are up-regulated, and the genes coding for the corresponding histone methyltransferases and histone acetyltransferases are also upregulated [44]. Annexin genes have been implicated in fin regeneration, and the annexin A2a (*anxa2a*) and A2b (*anxa2b*) genes are indeed upregulated during regeneration. H3K27me3 and H4K20me3 play critical roles in *anxa2a* and *anxa2b* gene regulation [44]. The promoters of the genes, which diminish after development and are reactivated during fin regeneration, contain the bivalent H3K4me3/H3K27me3 domains. During regeneration, the histone demethylase Kdm6b.1 is upregulated in the blastema and mediates the removal of repressive H3K27me3 to activate gene expression. The knockdown of Kdm6b.1 by morpholinos results in a pronounced decrease in the caudal fin regeneration in zebrafish larvae [45]. On the other hand, the histone lysine methyltransferase EZH2 mediates H3K27me3 methylation. In *ezh2*-deficient mutants, the spinal cord and caudal fin fail to regenerate properly, suggesting that Ezh2 might also be involved in the process of regeneration in zebrafish [46].

Histone acetylation has been reported to contribute to hair-cell regeneration. Treating zebrafish embryos with pharmacological inhibitors of the histone deacetylase (Hdac) trichostatin A or valproic acid decreases the number of hair cells that regenerate after injury [47]. Either the *hdac1* gene disruption or Hdac inhibitor treatment blocks the differentiation of liver progenitor cells into hepatocytes and biliary epithelial cells after hepatocyte ablation [48]. In retinal regeneration, Hdac1 inhibition suppresses the formation and differentiation of MGPCs, and results in compromised regeneration. Hdac1 is an essential regulator of the genes associated with retinal regeneration, such as *ascl1a*, *lin28a*, and *her4.1* [49]. Future studies need to pay more attention to profiling the global histone modifications and identifying the genes they affect during organ regeneration.

## 4. Role of the SWItch/Sucrose Non-Fermentable (SWI/SNF) Complex in Organ Regeneration

**Figure 2:**
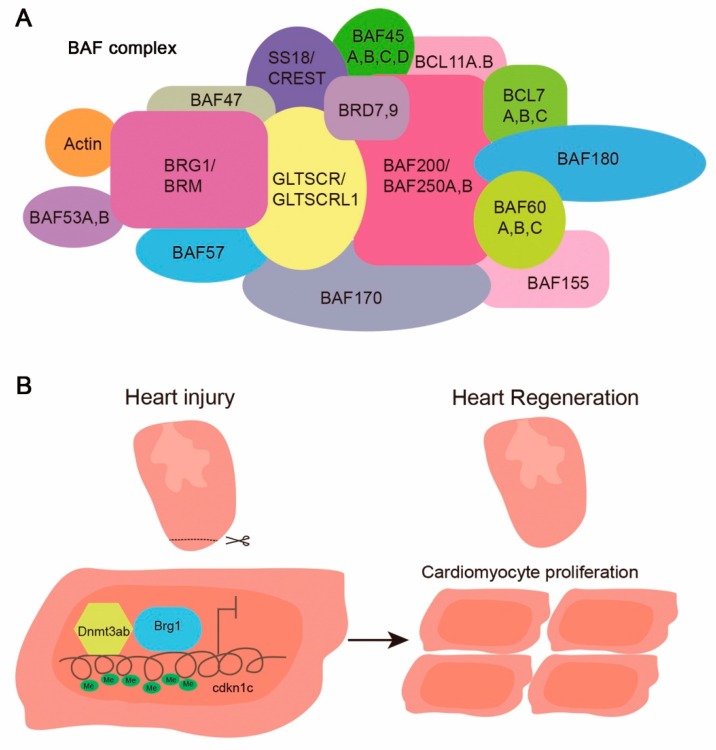
Composition of the mammalian SWI/SNF complex and Brg1 function in zebrafish heart regeneration. (**A**) Subunit composition of the mammalian SWI/SNF complex; (**B**) Cartoon showing how Brg1 regulates zebrafish cardiomyocyte proliferation and regeneration via a derepression mechanism. The injury-induced Brg1, via its interaction with Dnmt3ab, inhibits the expression of cdkn1c by increasing the methylation level of CpG sites at the cdkn1c promoter [61].

The SWItch/sucrose non-fermentable (SWI/SNF) complex is a protein complex with an ATPase core subunit encoded by either Brg1 or Brm. The mammalian SWI/SNF complex consists of a large number of subunits (as shown in Figure 2A), most of which have orthologue(s) in zebrafish, except CREST and BCL7C. This complex is recruited to the promoters of its target genes where it remodels the chromatin architecture in an ATP-dependent manner and alters the accessibility of chromatin to facilitate or inhibit gene transcription [50,51].

The SWI/SNF complex plays an essential role in mouse embryonic development, as follows: Brg1-null embryos die before implantation [52]; endocardial-specific knockout of Brg1 results in trabeculation defects [53]; in the endocardium, Brg1 is essential for the formation of ventricular cardiac jelly [53]; and Brg1 deletion in the myocardium results in decreased chamber size, impaired looping, and premature death in most mutant embryos before embryonic day 10.5 [54]. Mutant Brg1 in zebrafish also results in abnormal cardiac morphogenesis and patterning [54]. Epicardial Brg1 is essential for the development of epicardium-derived cells. It has been reported that epicardium-specific Brg1-knockout reduces the levels of coronary smooth muscle markers and the number of Wt1-positive cells [55]. Baf60c-knockout results in cardiac hypoplasia and dysfunction. Embryos derived from embryonic stem cells with Smarcd3 (baf60c) siRNA-mediated knockdown show defects in heart morphogenesis and embryonic death at embryonic days 10.0–11.0 [56]. The myocardial-specific deletion of Baf60c results in postnatal dilated cardiomyopathy with impaired contractile function [57]. The baf180-null heart has severely hypoplastic myocardial walls and ventricular septal defects [58]. Baf250a depletion results in the arrhythmic contraction of cardiomyocytes with a changed expression of cardiac-specific genes and cardiac transcription factors in cardiomyocytes induced from P19 cells [59]. Together, these results suggest that several subunits of the SWI/SNF complex are essential for heart development and maturation.

The SWI/SNF complex plays a fundamental role in stem-cell pluripotency and differentiation [60]. Baf60C has been identified as a key regulator of myogenic lineage commitment [56]. Working together with the other transcriptional factors Gata4 and Tbx5, Baf60c directs the differentiation of mouse mesoderm into beating cardiomyocytes, partly by permitting the binding of Gata4 with cardiac-specific genes [54].

Components of the SWI/SNF complex have been reported to regulate the proliferation of various cell-types via different molecular mechanisms. In hepatocytes, Brg1 recruits lysine demethylase 4 (KDM4) to activate the β-catenin target genes and to promote cell proliferation. Brg1 deletion in hepatocytes inhibits liver regeneration and dampens survival after partial hepatectomy in mice [62]. Mutations of the SWI/SNF complex subunits or proteins that interact with these subunits are found in ~20% of human cancers [63]. The knockdown of BRG1 promotes senescence in colorectal cancer cells by upregulating the p53 and p21 expression. BRG1 interacts with Sirtuin1, which deacetylates p53 at K382 and inhibits p53 expression [64]. It has also been reported that BRG1 interacts with MSP58 and p53 to modulate the expression of p21 [65]. In leukemia cells, BRG1 is required for the expression of Myc. The SWI/SNF complex resides on a lineage-specific enhancer 1.7 Mb downstream from Myc, and is required for long-range chromatin looping interactions with the Myc promoter [66]. BRG1 may bind with the pax7 promoter to activate pax7, which is required for the proliferation of the satellite cells in mouse myoblasts. Brg1-null myoblasts show reduced proliferation and increased apoptosis [67].

Brg1 is not only coordinated with transcription factors to regulate gene expression, it also controls gene expression at least partly by regulating histone H3K27-methylating and demethylating enzymes in medulloblastoma [68]. Brg1 also regulates the higher-order organization of the genome [69].

The SWI/SNF complex participates in cell proliferation and differentiation. It would not be surprising if Brg1 also participated in organ regeneration in zebrafish. After resection of the apex of the zebrafish heart, *brg1*, *baf60c*, and *baf180* are upregulated [61]. Their expression is induced at ~2 dpa, peaks at ~14 dpa, and decreases to the basal level at 30 dpa, when regeneration is completed. The upregulation of the *baf60c* expression after apical resection is also found in axolotls [70]. The overexpression of dominant–negative *brg1* blocks cardiac regeneration and leads to evident scar formation in zebrafish at 30 dpa. The cardiac-specific inhibition of *brg1* results in reduced cardiac proliferation. Mechanistically, Brg1 interacts with Dnmt3ab to suppress the expression of *cdkn1c* (cyclin-dependent kinase inhibitor 1c) by increasing the methylation of its promoter (Figure 2) [61]. Thus, Brg1 regulates myocardial proliferation and regeneration via interacting with DNA methyltransferase to down-regulate the expression of cell-cycle inhibitors [61]. Future studies need to address whether the over-expression of Brg1 and/or other subunits of the SWI/SNF complex are sufficient to promote the entry of mammalian cardiomyocytes into the cell cycle.

## 5. MicroRNA and Organ Regeneration

MicroRNAs (miRNAs) play important roles in epigenetic regulation. Mature miRNA binds to the 3′-untranslated region of the target mRNA, leading to either mRNA degradation or the inhibition of protein synthesis. A single miRNA can bind with and inhibit many target genes simultaneously, while one mRNA can be regulated by many miRNAs [71].

miRNAs are vital for normal cardiac development and function. The cardiac-specific deletion of Dicer, which is essential for cleaving pre-miRNA to form mature miRNA, causes poorly-developed ventricular myocardium and embryonic lethality at embryonic day 12.5 in mice, supporting an essential role of miRNA in heart development [72]. Both miR-1 and miR-133 are strongly and specifically expressed in the heart and skeletal muscle. The overexpression of either miR-1 or miR-133a results in thin ventricular walls, decreased cardiomyocyte proliferation, heart failure, and embryonic lethality in mice [73,74]. miR-1 and miR-133 also have distinct functions in cardiac and skeletal muscle development in *Xenopus* [75].

In zebrafish, miR-218a-1 and miR-218a-2 are located in an intron of *slit2* and *slit3,* respectively, and the SLIT receptors Robo1 and Robo2 are their predicted targets. MiR-218 morphants have delayed heart field migration and display pericardial edema and a morphologically abnormal heart at 48 h post-fertilization in zebrafish. They regulate vascular endothelial growth factor signaling and endocardial migration via the Slit/miR218/Robo axis [76]. MiR-218 overexpression leads to the expansion of *tie-2* expression, and a slower migration of cardiomyocytes [77]. Both miR-218 and miR-19a are regulated by Tbx (T-box) 5 [77,78]. miR-218 knockdown rescues the heart defects induced by the overexpression of Tbx5, while the miR-19a replacement is able to partially rescue the cardiac defects induced by *tbx5*-knockdown, suggesting that both miR-218 and miR-19 are important mediators downstream from tbx5 signaling [77,78]. MiR-138 is expressed in the zebrafish heart and is required for normal cardiac maturation and patterning, and its morphants induce evident abnormal cardiac looping, the ventricular expansion of genes that are normally restricted to the atrioventricular valve, and cardiac dysfunction [79]. The expression of miR-21 is essential for valve formation in the zebrafish heart [80], while miR-92 regulates endoderm formation and its overexpression causes cardia bifida [81].

miRNAs in cardiac regeneration: Changes of miRNA expression are found after cardiac injury in zebrafish. Some miRNAs such as let-7i, miR-21, miR-146a, and miR-204 are upregulated at 7 dpa compared with the uninjured samples, while other miRNAs are downregulated, including miR-92b, miR-150, miR-128, and miR-133 [82]. The overexpression of miR-133 restricts cardiomyocyte proliferation and inhibits myocardial regeneration after injury, while miR-133 depletion enhances cardiomyocyte proliferation [82]. MiR-101a is dynamically expressed after ventricular resection in zebrafish, being down-regulated at 3 dpa, then highly upregulated at 7–14 dpa, and returning to the basal level at 30 dpa when the regenerative process is complete. The depletion of the miR-101a expression promotes cardiomyocyte proliferation at 3 dpa, but sustained inhibition of its expression results in increased fibrosis. Mechanistically, *fosab* has been identified as the target gene of miR-101a during cardiac regeneration [83]. 

After amputation of the ventricular apex, miR-99/100 and let-7a/c are downregulated during regeneration. Accordingly, two predicted targeted genes of miR-99/100, *fntb* and *smarca5*, are significantly upregulated during heart regeneration. Administration of miR-99/100 mimics leads to a deficiency in zebrafish heart regeneration. Although the miR-99/100 and let-7a/c pathways are highly conserved between zebrafish and mammals during development, they fail to activate after cardiac injury in mammals. The expression levels of miR99/100, Let-7a/c, FNTB, and SMARCA5 remain constant before and after myocardial infarction in mice. The forced downregulation of miR-99/100 and Let-7a/c promotes the dedifferentiation and proliferation of cultured adult mouse cardiomyocytes. The inhibition of either miR99/100 or Let-7a/c results in improved cardiac function, increased cardiomyocyte proliferation, and reduced fibrotic scarring in a mouse model of myocardial infarction [84]. Similarly, miR-26a, which is the most abundant miRNA in zebrafish heart, is down-regulated after zebrafish ventricular resection, but remains constant in the mouse heart after ligation of the left anterior descending coronary. The predicted targets of miR-26a are strongly induced in the resected zebrafish heart, but not in the infarcted mouse heart. The inhibition of miR-26a by LNA (locked nucleic acid)-modified oligonucleotides against miR-26a transfection increases the proliferation of cultured cardiomyocytes, while the inhibition of miR-26a in neonatal mice significantly stimulates cell division [85].

The zebrafish heart is capable of full regeneration, but this capacity is lost in mammals one week after birth. Although miRNA clusters are evolutionally conserved between zebrafish and mammals, the transcriptional regulation of miRNA clusters is distinct. By comparing the profiling of miRNA and mRNA between zebrafish and mouse hearts upon injury, investigators report that a total of 45 miRNA-dependent networks are differentially regulated, but the underlying mechanisms remain largely unknown [85]. Future comparative studies of these species are needed in order to address the difference in the regenerative potential at the level of miRNAs in order to gain novel insights into the molecular mechanisms for improving mammalian heart regeneration. Although each miRNA may target multiple mRNAs, either the disruption or overexpression of miRNAs results in more specific and predictable gene regulation than those by interfering with other epigenetic regulators. A cluster of miRNAs have been reported to promote mouse and rat cardiomyocyte proliferation and cardiac repair in mice [86]. Because of the easy administration of miRNA mimics or anti-miRNA/miRNA sponges in vivo [87], miRNAs may provide new tools to trigger mammalian cardiac regeneration in animal models, and thus reveal new therapeutic targets for myocardial infarction in humans.

miRNAs in retinal regeneration: Dicer knock-down results in the reduced proliferation of Müller glia-derived neuronal progenitor cells, indicating that miRNAs are essential for retinal regeneration in zebrafish [88]. It has been reported that the Ascl1a/Lin-28/let-7 loop is essential for the regulation of the dedifferentiation of Müller glia. Lin-28 suppresses the let-7 expression, and in turn, Let-7 inhibits the expression of a group of regeneration-associated genes, such as *ascl1a*, *lin-28*, *oct4*, *pax6b*, and *c-myc*. Let-7 is associated with cellular differentiation, while Lin-28 is associated with proliferation [89]. In addition, Let-7 miRNA is required for the translational regulation of Shh signaling components, which are essential for MGPC induction and retinal regeneration [90]. Other studies have shown that miR-203 inhibits MGPC proliferation and is downregulated during retinal regeneration [91].

miRNAs in spinal cord regeneration: miR-133b is essential for locomotor recovery and axon regeneration in adult zebrafish [92], and can improve locomotor recovery after spinal cord injury in mice [93]. However, miR-133b expression in a single Mauthner cell inhibits axon regeneration in a model using two-photon axotomy in zebrafish larvae, and its inhibition promotes axon outgrowth via the regulation of *tppp3*, which can promote axon regeneration [94]. These different results on the function of miR-133b may be because of the use of different injury models, and need to be resolved in future studies. 

miRNAs in fin regeneration: miRNAs are also required for fin regeneration. It has been reported that the morpholino knockdown of dicer results in impaired fin regeneration. miR-203 inhibits the Wnt signaling transcription factor Lef1 and negatively regulates fin regeneration, whereas miR-203 knockdown results in elevated Lef1 levels and fin overgrowth [12]. Together, miR-203 and other miRNAs play essential roles during fin regeneration.

## 6. Perspective on Research Directions

In this review, we attempted to summarize the majority of recent findings on the epigenetic regulation of organ regeneration in zebrafish. Epigenetic modifications are among the regulatory machinery for gene activation and silencing at both transcriptional and translational levels. Accumulating evidence supports the idea that DNA methylation, histone modifications, the SWI/SNF complex, and noncoding RNAs have critical functions in adult organ regeneration. The logic of the actions by epigenetic regulation is not clear; for example, the temporal scale and spatial locations (cell types) remain to be addressed. With new developments in genome-editing, single-cell RNA-sequencing, and high-resolution live imaging technology, it is to be anticipated that spatiotemporal mapping of organ regeneration at a single-cell resolution will be achieved in the near future.

Given that that the zebrafish has an incredible capacity for regenerating most of its organs in adulthood, it has become a major model organism for studying organ regeneration. This field has rapidly moved forward so as to resolve the cellular sources of de novo regeneration, the underlying mechanisms that balance inflammation and fibrosis, and the molecular signaling pathways that regulate the entry of quiescent cells into the cell cycle, as well as beginning to address how regeneration is terminated properly. Emerging evidence suggests that epigenetic regulation is engaged in each of the above regenerative processes. In combination with a variety of epigenetic tools, such as chromatin immunoprecipitation-sequencing (ChIP-seq), assay for transposase accessible chromatin-sequencing (ATAC-seq), bisulfite-sequencing (BS-seq), and Hi-capture sequencing (HiC-seq), we expect to achieve the global profiling of epigenetic modifications in organ regeneration, and thus decipher more regenerative genes/factors and regeneration enhancers [95]. The field will continue to address how epigenetic complexes and organ-specific transcription factors are coordinated to fine-tune regenerative programs such as in reactivating organ progenitors/stem cells, or promoting dedifferentiation, proliferation, and differentiation. In addition, it remains largely unknown how organ regeneration is properly terminated in order to achieve the appropriate morphological and functional identity, as well as how fibrosis is generated and resolved during organ regeneration.

Investigating the molecular mechanisms of epigenetic regulation in organ regeneration in zebrafish will not only help to understand the basic science of the regenerative potential, but also may lead to new technologies and strategies for improving the regenerative capacity of mammals, including human beings. With the great advances in epigenetics across biomedical research at this time, epigenetic regulation is gradually moving onto center-stage in the field of de novo organ regeneration.
